# Helpful Factors in the Treatment of Depression from the Patient’s, Carer’s and Professional’s Perspective: A Concept Map Study

**DOI:** 10.1371/journal.pone.0167719

**Published:** 2016-12-16

**Authors:** Rosa A. van Grieken, Hanka F. Verburg, Maarten W. J. Koeter, Jessica Stricker, Udo W. Nabitz, Aart H. Schene

**Affiliations:** 1 Department of Psychiatry, Program for Mood Disorders, Academic Medical Center, University of Amsterdam, Amsterdam, The Netherlands; 2 Department of Research and Quality, Arkin Mental Health Care Services, Amsterdam, The Netherlands; 3 Department of Psychiatry, Radboud University Medical Center, Nijmegen, The Netherlands; 4 Donders Institute for Brain, Cognition and Behavior, Radboud University, Nijmegen, the Netherlands; IRCCS Istituto Auxologico Italiano, ITALY

## Abstract

**Objective:**

Depression research has resulted in knowledge about neurobiology, pharmacological strategies and short-term cost-effective treatments. However, more than two-thirds of all depressed patients experience insufficient improvement. Therefore, a better understanding of what patients, carers and professionals perceive as most helpful in the treatment of depression is needed.

**Methods:**

Concept mapping, a mixed-method design, was used to identify the patients (n = 33), carers (n = 22) and professionals (n = 50) perspectives. In six brainstorm sessions, the patients, carers and professionals generated 795 ideas, which were condensed into 55 unique statements. Subsequently, 100 participants prioritized and sorted these statements, which were analysed by multidimensional scaling and cluster analysis and visualized as a two-dimensional map.

**Results:**

The 55 statements were clustered in 10 factors and further grouped into four main-factors (meta-clusters): ‘Professional therapist’, ‘Treatment content’, *‘*Structured treatment process’ and ‘Treatment organisation’. Patients and carers prioritized ‘Treatment organisation’ higher than professionals, but overall there was considerable agreement about the factors of treatment the participants perceived as most helpful including factors related to the therapeutic relationship and the ‘creation of hope’.

**Conclusions:**

Our study identified factors of depression treatment perceived as helpful according to patients, carers and professionals. Findings suggest that in a scientific era with emphasis on biological psychiatry, not only patients and carers, but also professionals consider *aspecific* factors the most helpful. Further studies might show that factors we found to be helpful in the treatment for depression, can be generalized to mental health treatment in general.

## Introduction

In the current era with an emphasis on biological psychiatry and time-limited protocollized treatments, depression research has resulted in knowledge about neurobiological mechanisms, pharmacological strategies and cost-effectiveness of short-term treatments [1, 2]. However, it is well known that more than two-thirds of all depressed patients experience insufficient clinical improvement, despite the wide range of therapeutic approaches available today [1]. Insufficient therapeutic success may be due to the fact that too often interventions fail to take into account that patients prefer treatments in accordance with their personal ideas, goals and preferences [3]. More importantly, the patient’s perspective on the treatment of depression influences treatment outcomes [4, 5].

Knowing that major depressive disorder (MDD) is one of the most common psychiatric disorders with devastating effects both for those who suffer from it and their carers [6], it is important to have a better understanding of what patients perceive as most helpful in the treatments for depression. This knowledge is especially important because depressed patient’s perspectives about treatment often differ from those among professionals [7]. For example, findings from a systematic review suggest that patients prefer psychotherapy rather than the occasional professional advice for pharmacological treatment strategies [8]. Moreover, the role of the carer in depression research is relatively invisible, but invaluable, since they are the daily witnesses in perceiving how treatment affects the depressed patient. Although professional depression guidelines [9, 10] require the carer’s engagement into treatment, carers often feel frustrated about their exclusion from treatment [11].

In today’s real-world practice, professionals may want to focus on more than just the application of evidence-based protocollized approaches, since multiple factors contribute to the success of treatments [12]. As said, perspectives of professionals may differ from those of patients and carers, and so we studied in an integrative way what the three groups—patients, carers and professionals—perceive as helpful factors in the treatment of depression [13]. We identified the following research questions: 1) What are helpful factors in the treatment of depression from the patient’s, carer’s and professional’s perspective? 2) What factors do patients, carers and professionals perceive as most helpful? 3) What kind of differences exist in the perspectives of patients, carers and professionals regarding these factors? With this knowledge, professionals will have the potential to adjust the specific needs of depression treatment for each individual patient to develop personalized treatment, ameliorate shared goals, and consequently accomplish better treatment compliance and effectiveness.

## Methods

### Design

In order to answer these questions and based on earlier experiences in our group [14, 15], we found ‘concept mapping’ to be the most promising and adequate method. Trochim [16] developed this structured mixed method in order to organize diverse ideas into an understandable and coherent framework. Concept mapping involves advanced statistical analyses of qualitative data, for the conceptualisation of a specific, mostly complex, subject. This method is successfully applied in the field of health, social and management sciences [17, 18].

Moreover, the combination of both qualitative and quantitative analyses makes this method more data-driven than other qualitative research methods like in-depth interviews. Through the usage of group processes, joint discussion and exploration, this method allows the encouragement of participants to bring up more ideas than would appear in individual approaches like interviews. Moreover, concept mapping generates the conceptual framework by a statistical algorithm, which can be replicated by others. The internal validity of the approach is high and the findings allow identification of similarities and differences between various perspectives [17].

### Participants

Although there is no ‘correct’ number of participants when using concept mapping, and some argue that 20 participants in total is sufficient, we aimed for approximately 100 participants, a much larger group, to reach the maximum in terms of the exploration of ideas [18]. The study was presented to the Medical Research Ethics Committee of the Academic Medical Centre Amsterdam. In line with the Dutch legislation, this Committee decided that the study did not require extensive ethical review as participants were recruited on a volunteer basis and were not requested to undergo any incriminating intervention. Written informed consent was obtained from the participants.

#### Patients

Patients were eligible for the study if they met the following criteria: (a) at least one major depressive episode (MDE) according to DSM-IV-TR criteria at some point within three years preceding participation; (b) professional treatment for the most recent MDE (e.g. from a psychiatrist or a psychologist) and (c) currently in remission, as indicated by a score of less than seven on the Hamilton Depression Rating Scale (HDRS) [19], in order to minimize bias because of ‘negative MDE related cognitions’. Exclusion criteria were: age younger than 18 years; insufficient understanding of the Dutch language; a terminal disease; mental retardation; suicidality and bipolar-, psychotic-, anxiety- or cognitive disorder. Patients were recruited through a request for study participation by e-mail to members of the Dutch ‘Depression Association’.

#### Carers

Carers were included if they were partner, family member or friend of a patient who received depression treatment. We recruited carers by asking participating patients to contact their relations who were involved with their depression treatment.

#### Professionals

Professionals were included if they had substantial experience with treatment of depression for at least one year. We purposively sampled professionals with diverse professional backgrounds (e.g. psychiatrists, psychologists, occupational therapists and psychiatric nurses) by approaching several Dutch academic and non-academic mental health care organisations.

### Brainstorm sessions to generate statements

Concept mapping starts by defining the focus. In the current study, the participants were encouraged to brainstorm about their perspectives concerning the focus: ‘*Which factors of depression treatment do you perceive as helpful for recovery*, *from your experience?’* We carried out six different brainstorm sessions: patients and carers were mixed and equally divided over four sessions. The two sessions with professionals were held separately to ensure patients and carers could talk freely and without reservation. The number of participants of these six brainstorm sessions varied between 6 and 13 and each session took approximately 1.5 hours. All sessions were guided by trained researchers (RAvG, HFV and JS; respectively experienced as a psychiatric trainee, research fellow and occupational therapist).

We approached 90 patients, of whom 21 (23.3%) could be included and participated. Patients invited 22 carers. We invited 39 professionals, of whom 20 (51.3%) participated. Participants in the brainstorming sessions were not required to be the same participants who carried out the prioritizing and sorting assignment [18]. See Table 1 for all participants.

**Table 1:** Participants attending brainstorm sessions and prioritizing and sorting assignment.

Participants	Brainstorm sessions		Prioritizing and sorting	
	**n**^1^	**(%)**	**n**	**(%)**
Patients	21	(33)	31	(31)
Carers	22	(35)	19	(19)
Professionals	20	(32)	50	(50)
**Total**	**63**	**(100)**	**100**	**(100)**

^1^ Not all brainstorm participants did the prioritizing and sorting assignment and vice versa.

During the brainstorm sessions, all ideas generated around the focus were visualized for the participants via a digital projector. The collection of ideas was continued until no new ideas emerged (saturation).We used audiotape and full transcription of the sessions to catch the ideas in an accurate manner. Based on these transcriptions, the researchers (RAvG, HFV and JS) combined 795 ideas and removed ideas that overlapped or that were not related to our focus. A total of 256 unique ideas remained. Next, ideas were further condensed by combining similar ideas, e.g. ‘the clinician should involve carers’ and ‘to look for a solution in treatment together with carers’, to formulate the quality demand statements with an abstraction level that was neither too specific nor too general. Consensus meetings between members of the research group led to a final set of 55 unique statements.

### Prioritizing and sorting the statements

These 55 final statements were each printed onto cards and sent to the participants as a homework assignment. For this stage, we approached 76 patients, of whom 31 (34.4%) participated. Of the 22 carers invited, 19 participated. We invited 53 professionals, of whom 50 (94.3%) participated (see Table 1). We invited more participants than only the ones in the first stage. In the prioritizing assignment, each individual participant had to prioritize the 55 statements by dividing them into 5 groups of equal size. Group 1 was defined as ‘least helpful’ in depression treatment and group 5 as ‘most helpful’.

In the sorting assignment, each participant was asked to sort the 55 statements based on their own meaning of whether they belonged to the same category. Participants were asked to use between 2 and 12 and each category had 2 to 20 statements.

### Statistical analyses

The prioritizing and sorting data from all participants were analysed using the computer programme ‘Ariadne’, designed to support concept mapping [20–22]. First, the multidimensional scaling analyses positioned the statements as points in a two-dimensional map: ‘point map’. The point map showed the typical circumplex distribution of the statements, in which the distance between the statements represents how often the participants sorted them in groups.

Second, the positioned statements on the point map were clustered, the ‘cluster map’, based on a hierarchical cluster analysis [18]. Subsequently, the relative importance of the statements and differences between the three participant groups were calculated based on the participant’s prioritizing score. Univariate analysis of variance (ANOVA) and multiple comparison tests of the mean priority of the clusters and statements were carried out. The mean cluster score was defined as the sum of all statements of the cluster. These data were analysed using SPSS (version 21) for Windows.

## Results

Table 2 displays the demographics of each participant group and shows that participating carers were partners (80%), family members (10%) and friends (10%). Participating professionals were psychiatrists (27%), psychologists (28%), psychiatric nurses (30%) and creative or psychomotor therapists (5%). Seventy percent of these professionals had more than 5 years of experience with depression treatments. Of all professionals, 52% worked for different regional mental health care institutions and 48% were academic professionals. Patient characteristics are shown in Table 3.

**Table 2:** Demographics of patients (PA), carers (CA) and professionals (PR).

	PA (n = 33)	CA (n = 22)	PR (n = 50)	Total (n = 105)^a^
Gender (% men)	18.2	63.6	40	38.1
Age (years)				
• 20–29 years	0	1	4	5
• 30–59 years	24	17	41	82
• 60+ years	9	4	5	18
Nationality (n)				
• Dutch	33	21	49	103
• Greek	0	1	0	1
• German	0	0	1	1

^a^ Total amount of participants of brainstorm sessions, prioritizing and sorting.

Five participants who attended the brainstorm sessions did not carry out the prioritizing and sorting assignment.

**Table 3:** Patient demographic and clinical characteristics (n = 33).

**Demographic characteristics**	**%**
Relational status	
• Single/separated	78.8
• Married/partnership	21.2
Educational level	
• Low (primary school)	3.1
• Intermediate (secondary school)	40.6
• High (college or university)	56.3
Employment	
• Unemployed	28.1
• Employed < 20 hrs/week	37.5
• Employed > 20 hrs/week	34.4
**Clinical characteristics**	
Number of depressive episodes	
• Single episode	9.1
• Recurrent (average # of episodes)	90.9 (5.9)
Last depressive episode	
• < 1 year	42.4
• 1–5 years	30.3
• > 5 years	27.3
Type of treatment history^a^	
• Psychotherapy	90.9
• Pharmacotherapy	97.0
• Psychomotor and/or creative therapy	36.4
• Other	27.3
Treatment setting^a^	
• Inpatient	21.2
• Daypatient	48.5
• Outpatient	75.8

^a^ Patients received different types of treatment or settings

### Cluster map

**Fig 1:**
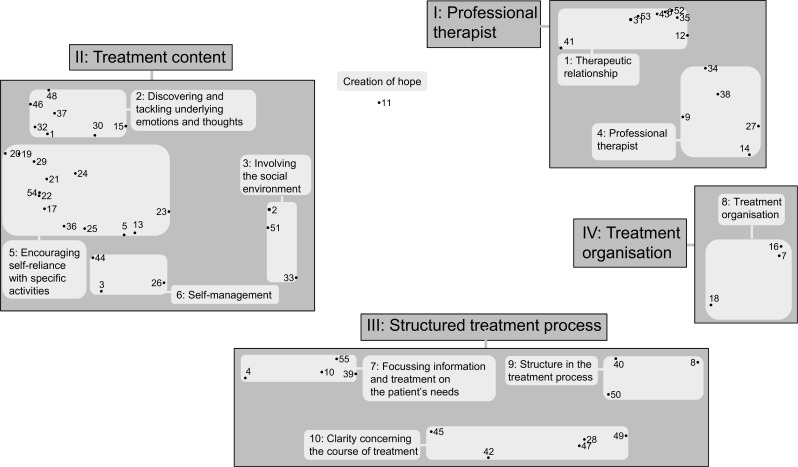
Cluster map: Helpful depression treatment factors according to patients, carers and professionals: statements, clusters and meta-clusters. The map displays the 55 statements presented as dots, the 10 clusters (1–10) and 4 meta-clusters (I-IV).>

The hierarchical cluster analyses resulted in a ten-cluster (1–10) solution and one single statement (ST 11): see Fig 1 for the cluster map. The proximity of the 55 statements in the cluster map is based on which statements were more likely to have been sorted in the same category based on their meaning according to the participants. The ten-cluster solution was considered the best interpretable one by consensus of all researchers according to the content of the statements belonging to these clusters. The circular distribution of the statements with its empty centre shows that there is little ambiguity about the positioning among participants: they agree about the reciprocal relation of the statements [18].

Additional meaning about their interrelation appeared when we combined these ten clusters into four meta-clusters. We numbered the (meta-)clusters in order of their perceived importance according to all participants. For each cluster we formulated a descriptive name based on the content and importance of the statements comprising the cluster [18]. An overview of the clusters with their corresponding statements, sorted by participant’s priority score, is presented in Table 4. We had the translation of the Dutch statements and (meta-) cluster names performed by a British native speaker.

**Table 4:** Meta-clusters, clusters and statements and the priorities of patients (PA), carers (CA) and professionals (PR).

(Meta)clusters with statements			Mean all parti-cipants	Mean PA	Mean CA	Mean PR
**Meta-cluster I: Professional therapist**						
**Cluster 1**	**Therapeutic relationship**		**3.18**	**3.38**	**3.11**	**3.08**
31	A therapist who is trusted by the patient		4.37	4.61	4.21	4.28
6	An understanding therapist		3.80	4.16	3.63	3.64
53	To have a good connection and feeling between patient and therapist		3.79	4.10	3.74	3.62
43	A therapist who addresses the patient’s talents and strengths		3.09	3.03	2.95	3.18
52	A therapist who, if necessary, can be provocative or confrontational		3.02	2.90	3.47	2.92
12	A flexible therapist who dares to think and act outside the box		2.69	2.94	3.11	2.38
41	The possibility of humour and lightness during the therapy		2.64	2.77	2.53	2.60
35	A therapist who acts as the patient’s ally		2.01	2.48	1.26	2.00
**Cluster 4**	**Professional therapist**		**2.97**	**3.09**	**3.06**	**2.87**
9	A therapy in which the patient is taken seriously		4.15	4.29	4.00	4.12
38	A therapist who keeps to his/her appointments		3.58	3.77	3.68	3.42
27	A therapist who prepares well and knows the patient’s file		3.16	3.48	3.53	2.82
34	A therapist who temporarily takes over control *(if necessary)*		1.99	2.00	1.79	2.06
14	A therapist who briefly repeats at the start of each session what was discussed the previous session		1.99	1.90	2.32	1.92
**Meta-cluster II: Treatment content**						
**Cluster 2**	**Discovering and tackling underlying emotions and thoughts**		**3.00**	**2.87**	**2.85**	**3.14**
1	To research behavioural patterns within a practical therapy and subsequently learning new behaviour		3.53	3.23	3.26	3.82
48	To pay attention to positive and success experiences		3.51	3.06	3.53	3.78
32	To research the negative self-awareness (sense of self) and subsequently learn alternative, more realistic thoughts		3.46	3.26	3.16	3.70
46	To handle specific avoidance behaviour of painful and difficult situations (for example regarding social contacts or administrative or financial problems)		3.22	2.68	3.05	3.62
37	To decrease feelings of powerlessness and guilt by drawing up complicated situations to enable the patient to grasp the situation		2.83	2.97	2.63	2.82
15	To pay attention to actively expressing suppressed feelings during treatment		2.55	2.77	2.53	2.42
30	To have a support which helps the patient to accept the depression		1.92	2.13	1.79	1.84
**Cluster 3**	**Involving the social environment**		2.99	3.01	3.12	3.93
2	A social environment which takes the depression into account		3.48	3.84	3.74	3.16*
51	To involve the social environment in the therapy		3.15	2.81	3.37	3.28
33	A therapy which offers help, guidance, explanation and support to the social environment of the patient		2.35	2.39	2.26	2.36
**Cluster 5**	**Encouraging self-reliance with specific activities**		2.95	2.88	2.98	2.98
21	Physical activity		3.80	3.48	3.42	4.14*
17	Learning how to recognize the first symptoms of the depression and develop a plan how to handle it in that situation		3.51	3.77	3.79	3.24
54	To create a daily schedule		3.49	3.19	3.58	3.64
25	To settle the patient’s sleeping rhythm (with medication if necessary)		3.47	3.39	2.95	3.72
20	To be stimulated to show initiative		3.37	2.74	3.58	3.68
24	To pay attention to acceptation of the depression		2.94	3.10	3.32	2.70
29	A patient’s realisation that he/she follows it for his/her own good and has to work at it		2.91	2.58	3.05	3.06
19	To find a reason to continue and be a part of society		2.88	2.55	2.84	3.10
23	To contact fellow sufferers		2.84	3.19	2.74	2.66
5	To assess additional practical problems and receive support or treatment for these		2.82	2.52	2.26	3.22
13	Researching the cause of the depression		2.81	3.10	3.26	2.46
36	To pay attention to nutrition		1.80	1.81	2.11	1.68
22	To keep a diary during the treatment period		1.68	1.97	1.89	1.42
**Cluster 6**	**Self-management**		**2.94**	**3.03**	**2.82**	**2.92**
3	To receive information about how a patient can work on his/her own recovery		3.65	3.68	3.79	3.58
26	To clearly postulate goals (short and long term) and to work towards these in small steps, to experience success		3.52	3.29	3.21	3.78
44	To read a book which offers insight to depression		1.64	2.13	1.47	1.40
**Meta-cluster III: Structured treatment process**						
**Cluster 7**		**Focussing information and treatment on the patient’s needs**	**2.94**	**2.75**	**2.89**	**3.08**
55		The right type and dosage of medication, which is adjusted to the specific patient’s needs	3.88	3.71	3.68	4.06*
10		Good education about medication	3.29	3.03	3.42	3.40
39		To receive a sound explanation on all aspects of depression	3.14	2.81	3.11	3.36
24		To follow a therapy on the internet	1.45	1.45	1.37	1.48
**Cluster 9**		**Structure in the treatment process**	**2.92**	**2.89**	**3.04**	**2.90**
8		Continuity within therapy	3.80	3.90	4.11	3.62
50		To regularly evaluate the therapy based on the patient’s experience	2.92	2.68	2.53	3.22
40		To provide an environment during therapy which feels welcoming and pleasant	2.05	2.10	2.47	1.86
**Cluster 10**		**Clarity concerning the course of treatment**	**2.92**	**2.86**	**3.04**	**2.92**
49		A therapy which quickly starts after the intake *(no waiting lists)*	3.70	4.03	4.37	3.24*
47		Therapist and patient establishing a clear therapy plan at the beginning of the therapy	3.27	2.74	3.37	3.56
28		Good follow-up care	2.78	2.81	2.68	2.80
45		To be able to follow a therapy with a variety of therapeutic approaches	2.75	2.90	2.68	2.68
42		To receive clear information when starting therapy in a brochure or via internet	2.11	1.81	2.11	2.30
**Meta-cluster IV: Treatment organisation**						
**Cluster 8**	**Treatment organisation**		**2.94**	**3.11**	**3.16**	2.76*
7	The possibility to have contact with the therapist at short notice		3.49	3.94	4.16	2.96*
16	Therapists who work well together		3.03	2.94	3.37	2.96
18	Therapist offers an alternative therapy or a referral when the therapy is not successful		2.31	2.45	1.95	2.36
**Single statement not included in a (meta-)cluster**						
11	Creating hope during treatment		3.63	3.65	2.84*	3.92

* Significant value (p<0.05).

#### Meta-cluster I

‘Professional therapist’, contains 13 statements grouped into two clusters: *1*. *Therapeutic relationship*: statements in this cluster all emphasize aspects that make the relationship between the participants meaningful; and *4*. *Professional therapist*: statements imply that patients and carers feel taken seriously when the therapist’s attitude is professional.

#### Meta-cluster II

‘Treatment content’, contains 25 statements grouped into four clusters: *2*. *Discovering and tackling underlying emotions and thoughts*: comprising statements focus on technical factors of treatment; *3*. *Involving the social environment*: statements emphasize the involvement of carers within treatment; *5*. *Encouraging self-reliance with specific activities*: statements all comprise factors that increase self-reliance; and *6*. *Self-management*: this cluster contains three statements that emphasize patients to work on their own recovery.

#### Meta-cluster III

*‘*Structured treatment process’, contains 12 statements grouped into three clusters: *7*. *Focussing information and treatment on the patient’s needs*: statements indicate that a careful exploration of the patient’s need for information about the various aspects of depression treatment is helpful; *9*. *Structure in the treatment process*: statements imply that a clear structure within treatment is perceived as helpful; and *10*. *Clarity concerning the course of treatment*: statements refer to the wish to be well informed about what the course of treatment looks like.

#### Meta-cluster IV

‘Treatment organisation’, contains three statements grouped into one single cluster: *8*. *Treatment organisation*: statements focus on organisations factors.

Finally, because the statistical analysis placed the single statement ‘Creating hope during treatment’ (ST 11) in the center of the cluster map, this statement has to be regarded as unique and helpful; it was not, or only minimally related to any broader category according to the participants.

### Differences between patients, carers and professionals

As Table 4 shows, the three participant groups generally agreed on which clusters they perceived as most helpful, as mean priority scores are aligned with each other. However, patients and carers perceived the cluster ‘Treatment organisation’ (CL 8) significantly more helpful compared to professionals (p<0.05). In addition, we explored differences between the three groups considering single statements. We took the top 10 statements perceived as most helpful as these were prioritized by the patients (see Table 5). Patients’ and carers’ top 10 appeared to be similar except for one statement (ST 7). The statements ‘a social environment which takes the depression into account’ (ST 2), ‘the possibility to have contact with the therapist at short notice’ (ST 7) and ‘a therapy which quickly starts after the intake’ (ST 49) were considered significantly more helpful by patients compared to professionals.

**Table 5:** Top 10 statements perceived as most helpful for depression treatment by patients (PA) and the differences compared to carers (CA) and professionals (PA).

Number and Statement		PA (n = 31)		CA (n = 19)		PR (n = 50)	
		Mean	*r*^1^	Mean	*r*	Mean	*r*
31	A therapist who is trusted by the patient	4.61	1	4.21	2	4.28	1
9	A therapy in which the patient is taken seriously	4.29	2	4.00	5	4.12	3
6	An understanding therapist	4.16	3	3.63	12	3.64	12
53	To have a good connection and feeling between patient and therapist	4.10	4	3.74	9	3.62	16
49	A therapy which quickly starts after the intake *(no waiting lists)*	4.03	5	4.37	1	**3.24**^**2**^	24
7	The possibility to have contact with the therapist at short notice	3.94	6	4.16	3	**2.96**	31
8	Continuity within therapy	3.90	7	4.11	4	3.62	14
2	A social environment which takes the depression into account	3.84	8	3.74	8	**3.16**	28
17	Learning how to recognize the first symptoms of the depression and develop a plan how to handle it in that situation	3.77	9	3.79	7	3.24	23
38	A therapist who keeps to his/her appointments	3.77	10	3.68	10	3.42	19

^1^ r: Rank order of statements.

^2^ Significant values (p<0.025) in bold.

## Discussion

In this study we were able to identify and describe 55 different, helpful factors in the professional treatment of depression from the combined patients’, carers’ and professionals’ perspectives. These 55 factors can be summarized into four meta-clusters: ‘Professional therapist’, ‘Treatment content’, ‘Structured treatment process’ and ‘Treatment organisation’. Interestingly, the clusters we found to be helpful were considered as almost of equal importance among patients, carers and professionals. The three groups only differed on one cluster (‘Treatment organization’) with patients and carers rating this higher than professionals. Findings suggest that, in a treatment era with emphasis on biological psychiatry and short term protocolled treatments, patients, carers and professionals still perceive what has become known as *aspecific factors* as the most helpful in the current psychiatric treatment of depression.

Our results about *aspecific* factors perceived to be helpful are not only in line with our previous study, in which we found similar *inhibiting* factors of depression treatment that are often not addressed in clinical practice [23], but it also reminds us of Lambert’s common factor theory [24, 25]. Common factors (e.g. therapeutic alliance and hope) account for a major part of the effectiveness of psychotherapy treatment, and that only 15% of treatment outcome is related to technical factors [24, 26, 27]. He discovered four primary factors that influence positive treatment outcome and calculated the relative importance of each factor, which are very similar to our findings: 40% of therapeutic change comes from extra-therapeutic factors (in our results represented by the meta-clusters ‘structured treatment process’ and ‘treatment organization’), 30% from therapeutic relationship (‘professional therapist’), 15% from hope and expectancy (‘creating hope during treatment’) and 15% from therapeutic technique (‘treatment content’).

Lambert’s findings result from an extensive literature review of over 40 years of psychotherapy outcome studies in another era, whilst our study uses a structured empirical method and includes the perspectives of patients, carers and professionals of depression treatment. This finding suggests that, despite the numerous studies and randomized controlled trials that have been conducted in the last decade on depression and treatment, patients, carers and professionals still perceive the therapeutic relationship, hope and other *aspecific* factors, that are not easy to ‘catch’ in research with quantitative bounders, to be the most important.

However, our results also show some *specific* factors to be important. For example, the *status apart* statement ‘creating hope during treatment’ (ST 11) was perceived as unique and very helpful in the treatment of depression, especially according to patients and professionals. Patient’s hope and expectations have long been considered a key ingredient of successful psychotherapy [24] and a more recent meta-analysis by Constantino [28] demonstrated positive effects of patients’ expectations on their treatment outcomes. Moreover, the loss of hope has been described as a cause of treatment resistance in depression [29]. Although many psychotherapies include elements that address various expectations, such strategies are rarely emphasized in scientific literature or clinical practice [30]. Furthermore, treatment guidelines for depression remain relatively silent about this complex *aspecific* factor [9, 10]. This result indicates that the factor hope has to be explored more profoundly, to discover the meaning and usage of hope in treatment.

It might be simplistic to relate decisions for treatment to perspectives only, because treatment for depression is a complex process where a number of variables must be taken in account, such as severity, comorbidity and patient’s history [31]. However, preferences are strongly associated with particular outcomes such as entry into treatment and development of the therapeutic alliance [32]. Furthermore, supporting preferences as part of depression treatment result in more patients receiving the treatment that is most suitable to them [8]. Further initial evidence [33] supports the idea that patients who are able to gain control over their treatment decisions may experience improved outcomes. Even though the participant’s perspectives about helpful treatment factors might not result in objective remission, according to our results it is obvious that clinical judgment should stop concentrating on drugs as the only ‘cure’ of the disease [12] and should enlighten perspectives to improve e.g. patient treatment commitment and support from carers.

### Strengths and limitations of the study

The main strengths of our study are the empirical exploration of three different participant perspectives, and the use of both qualitative and quantitative methods which offered the opportunity to address the experiences regarding all factors of the treatment of depression, from aspecific to specific. Our study also has some limitations. First, the concept map is a result of the three participant groups combined. Therefore, certain caution is necessary in comparisons of statement priorities and ranking of the different clusters between the patients, carers and professionals, due to the differences in the amount of participants within each group. Second, perspectives of participants are inherent to subjectivity. However, we aimed to explore the different perspectives, which is typical of qualitative research. Third, there may be different, helpful treatment approaches during different stages of a depressive episode, from mild to severe, and to specific (cultural) subgroups. Also, the participants were ethnic Dutch adults. Therefore, the perspectives are generalizable to adults from countries comparable to the Netherlands in terms of population and (mental) health care system. Future studies might include participants belonging to specific subgroups to specify helpful treatment factors. Fourth, we translated the Dutch statements and (meta-)cluster names to English and this might have resulted in slightly different meanings, however, we purposely selected a native British physician to minimize the possibility of bias in the meaning of the words.

The knowledge about the patient’s, carer’s and professional’s perspectives is a first step; further work needs to be done. For example, the development of a questionnaire to identify patient’s treatment perspectives and guidance for their carers, all in cooperation with the professional. This may help professionals to personalize treatment [34] and may contribute to higher levels of patient satisfaction and thereby, could make treatments more effective. Next, more research is needed to find out about if effectiveness really occurs if the patient’s, carer’s and professional’s perspectives are optimally used in treatment. Moreover, the factor ‘hope’ needs to be explored to discover the meaning and usage of this appearing new factor in treatment.

We believe that our findings broaden the view of clinical practice and clarify the importance of ‘common factors’ in psychotherapy research and real-world practice. We also believe that the implementation of our results in clinical practice might considerably improve a patient’s and carer’s engagement to treatment and better treatment outcome. Because of some overlap of our results with other research [25], further research might show that the factors we found to be perceived most helpful in the treatment for depression treatment can be replicated and generalized to mental health treatment in general.
